# Dichroic Optical Diode Transmission in Two Dislocated Parallel Metallic Gratings

**DOI:** 10.1186/s11671-018-2818-5

**Published:** 2018-12-04

**Authors:** Pengwei Xu, Xuefeng Lv, Jing Chen, Yudong Li, Jun Qian, Zongqiang Chen, Jiwei Qi, Qian Sun, Jingjun Xu

**Affiliations:** 1 MOE Key Laboratory of Weak Light Nonlinear Photonics, Tianjin Key Laboratory of Photonics and Technology of Information Science, School of Physics, Nankai University, Tianjin, 300071 China; 2 Collaborative Innovation Center of Extreme Optics, Shanxi University, Taiyuan, 030006 Shanxi China; 3 College of Environmental Science and Engineering/Sino-Canada R&D Center on Water and Environmental Safety, Nankai University, Tianjin, 300071 China

**Keywords:** Dichroic optical diode, Metallic gratings, Diffraction, Surface plasmons

## Abstract

An optical diode structure with two dislocated parallel metallic gratings is proposed and investigated numerically. Dichroic optical diode transmission is realized in this structure, i.e., optical diode effect is observed in two wavebands corresponding to inverse transmission directions. In the structure, two parallel metallic gratings with different grating constants are separated by a dielectric slab in between. The first illuminated grating acts as a selector for exciting surface plasmons at a proper wavelength. The other grating acts as an emitter to realize optical transmission. When the incident direction is reversed, the roles of two gratings exchange and surface plasmons are excited at another wavelength. In dichroic transmission wavebands, the optical diode structure exhibits extraordinary transmission and possesses high optical isolation up to 1. Furthermore, the operating wavebands can be modulated by changing structure parameters.

## Introduction

Optical diode, which transmits photons toward one direction and forbids the transmission in the reverse direction, has attracted considerable attention by virtue of the unidirectional transmission property [1]. Optical diode phenomena can be observed when time-reversal symmetry of light-matter interaction is broken. External magnetic field [2], bias voltage [3], acoustic wave [4], or time-dependent modulation [5, 6] can be applied to achieve the optical diode effect. In addition, the structure of spatial inversion symmetry breaking is an alternative choice, such as asymmetric multilayer structures [7], asymmetric photonic crystals [8], and asymmetric gratings [9]. In recent decades, metallic micro-nano structures gained great interest due to the promising properties of surface plasmons (SPs). Plasmonic devices are proposed in many research fields such as metasurface holography [10–14], refractive index sensor [15, 16], and filter [17, 18]. Plasmonic devices can strongly modify the interaction of electromagnetic fields in nanoscale [19]. The modulation on SPs can be realized through changing the surrounding dielectric environment and geometric parameters of metallic structures [20, 21]. Optical diodes composed of nanoscale metallic structures, for example, plasmonic layer sandwiched gratings [22, 23], cascaded plasmonic gratings [24, 25], plasmonic nanoholes [26], plasmonic slot waveguide [27], and plasmonic nanoparticle aggregates [28], are widely investigated for the purpose of optical information processing.

In this paper, dichroic optical diode transmission is obtained in two dislocated parallel metallic gratings sandwiching a dielectric slab. Both transmission enhancement and high isolation contrast ratio are achieved in the two operating wavebands with reverse transmission directions, because metallic gratings consisting of narrow slits exhibit extraordinary light transmission [29, 30] and asymmetric structures realize unidirectional transmission [27–31]. According to the illuminated order, two metallic gratings with different grating constants act as a selector and an emitter respectively. The selector selects the resonance wavelength by exciting SPs and, with the contribution of SPs, the emitter realizes light transmission. When the incident direction is reversed, the roles of two gratings exchange and SPs are excited at another wavelength. Therefore, the dichroic optical diode transmission is obtained. The thickness of the optical diode structure proposed in this paper is as small as 160 nm. With the development of nanofabrication technologies, many methods can be applied to the fabrication of metallic gratings structures, such as ultraviolet nanoimprint lithography [32], laser-direct-writing lithography [33], and electron-beam lithography [34]. The optical diode character is independent of the incident intensity. These properties imply that our structure has extensive potentials in optical integration.

## Methods

**Fig. 1:**
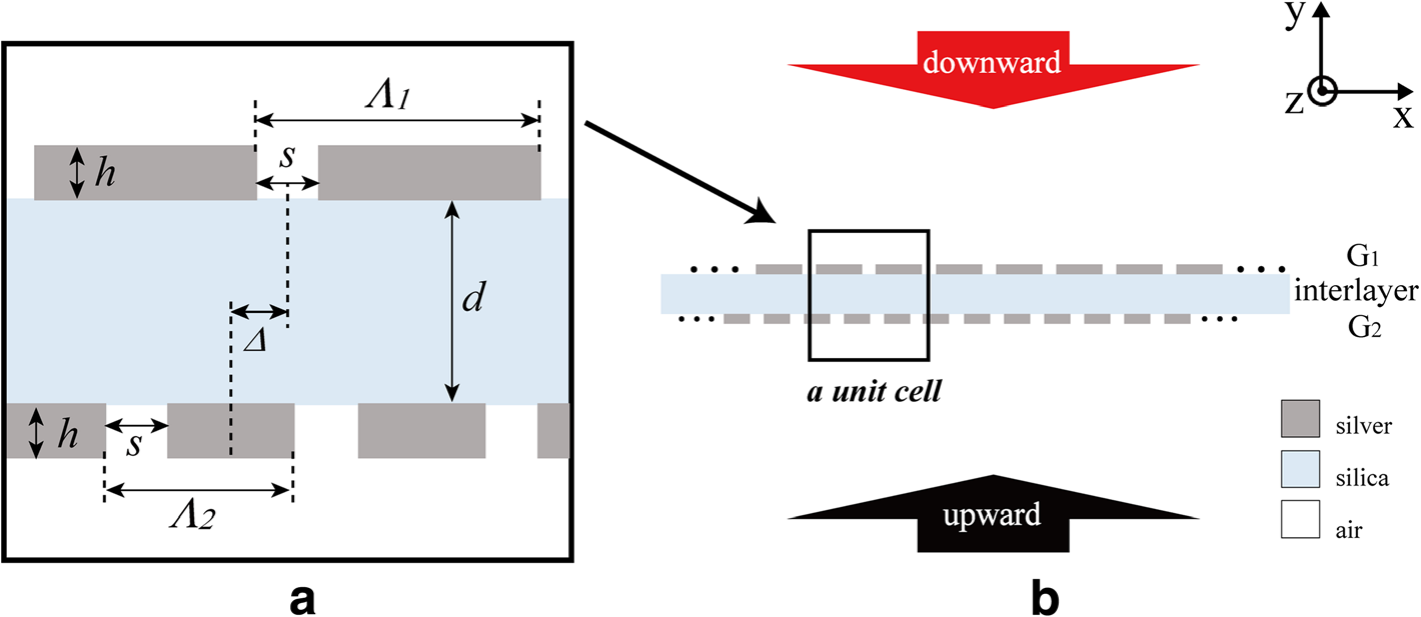
Schematic diagram of optical diode structure. **a** The unit cell. **b** Overall view

The scheme of optical diode structure is shown in Fig. 1. The structure consists of two silver gratings *G*_1_ and *G*_2_ sandwiching a silica layer. The thickness of silica layer is denoted as *d*. *G*_1_ and *G*_2_ have the same slit width *s*, the same thickness *h*, and different grating constants *Λ*_*i*_ (*i* = 1, 2). The structure is translational symmetric and the unit cell contains 2 units of *G*_1_ and 3 units of *G*_2_. *Δ* denotes the lateral relative position of *G*_1_ and *G*_2_ in a unit cell. Drude model [35] is used to describe the dielectric function of silver. The refractive index of silica is 1.5, ignoring its dispersion. The surrounding dielectric is air and its refractive index is 1. Normal incident plane wave of *p*-polarization is employed to investigate the optical diode effect.

Transmittance *T* of the optical diode structure is defined as follows:

1 $$ T=\frac{p_o}{p_i}, $$

where *P*_*i*_ is the incident power and *P*_*o*_ is the output power. *T* is simulated numerically by using finite-difference time-domain (FDTD) method [36]. Periodic boundary conditions are employed to the left and right sides, and perfect matching layer boundaries are applied to the top and bottom sides of our simulation model. *T*_*D*_ and *T*_*U*_ represent the transmittance for downward incidence and upward incidence, respectively. The optical diode property is described by isolation contrast ratio *η*:

2 $$ \eta =\frac{\left|{T}_D\hbox{-} {T}_U\right|}{T_D+{T}_U}. $$

Hence, *η* = 1 means the best optical diode performance.

### Results and Theoretical Analyses

**Fig. 2:**
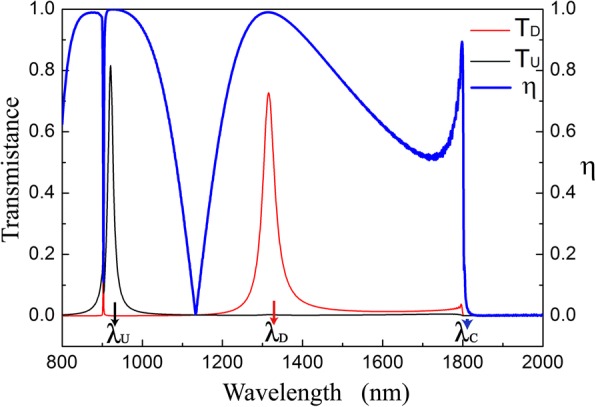
Transmission spectra and the isolation contrast ratio of the optical diode structure with *d* = 200 nm, *s* = *h* = 50 nm, *Λ*_1_ = 900 nm, *Λ*_2_ = 600 nm, and *Δ* = 0 nm

The transmittance and isolation contrast ratio of the optical diode structure are shown in Fig. 2. *T*_*D*_ is different to *T*_*U*_ when the incident wavelength is smaller than *λ*_*C*_. *T*_*D*_ reaches the maximum value 0.73 and *T*_*U*_ is 3.7 × 10^−3^ at *λ*_*D*_ (1315 nm). Whereas *T*_*U*_ reaches the maximum value 0.82 and *T*_*D*_ is 3.6 × 10^−4^ at *λ*_*U*_ (921 nm). The isolation contrast ratios at *λ*_*D*_ and *λ*_*U*_ are 0.990 and 0.999, respectively. Figure 2 shows that optical diode effect is obtained at around *λ*_*D*_ and *λ*_*U*_, and the two wavebands have reverse transmission directions. In the dichroic diode operating wavebands, the structure exhibits extraordinary transmission.

**Fig. 3:**
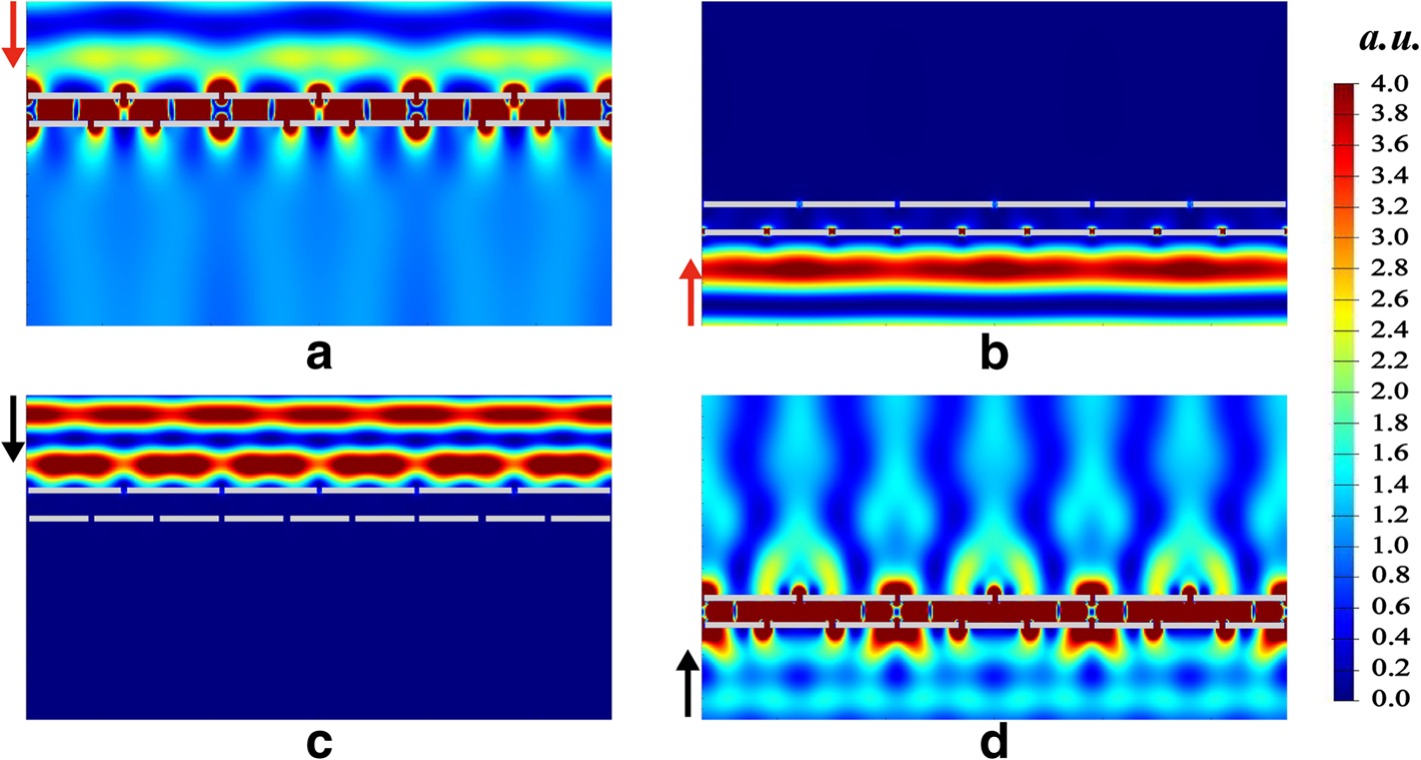
Distributions of electric field intensity |*E*|^2^ for downward incidence at *λ*_*D*_ = 1315 nm (**a**), upward incidence at *λ*_*D*_ = 1315 nm (**b**), downward incidence at *λ*_*U*_ = 921 nm (**c**), and upward incidence at *λ*_*U*_ = 921 nm (**d**)

In order to understand the dichroic optical diode transmission, the electric field intensity |*E*|^2^ at two operating wavebands are simulated. As shown in Fig. 3a, d, electric field is enhanced between two gratings when light transmits through the optical diode structure. Meanwhile, Fig. 3b, c show the reverse blocking status. The enhancement of electromagnetic field between two gratings is due to the SPs at two adjacent silver/silica interfaces. The types of SPs at two gratings are different, which are classified as structured SPs (SSPs) and induced SPs (ISPs) respectively. SSPs is excited and generates at the first illuminated grating (selector). ISPs is induced at the latter grating (emitter) by the coupling between SPPs and the adjacent silver/silica interface. Due to SSPs and ISPs, light transmits through the optical diode structure.

**Fig. 4:**
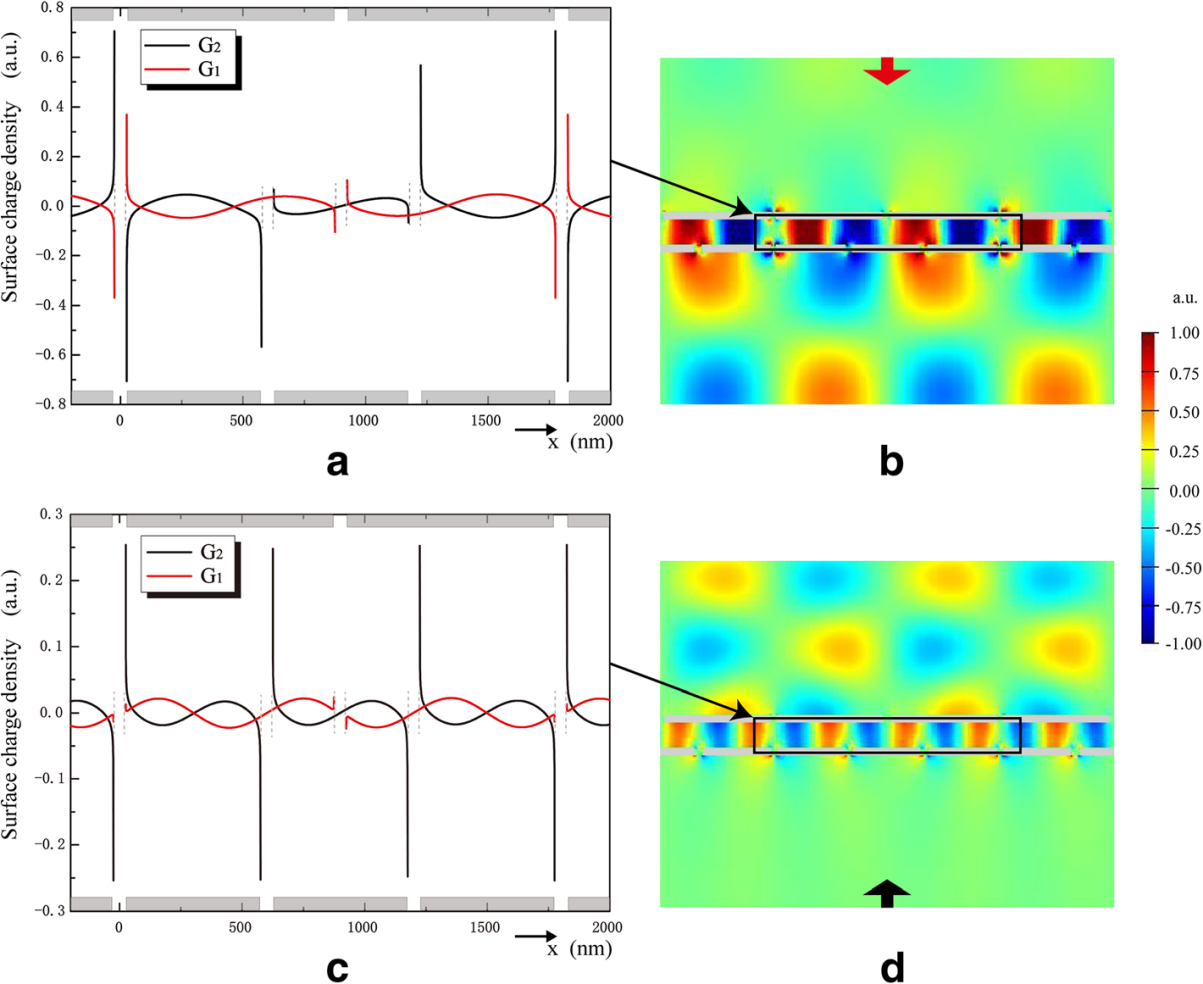
The surface charge density on the silver/silica interface at *G*_1_ and *G*_2_, under the condition of downward incidence at *λ*_*D*_ = 1315 nm (**a**) and upward incidence at *λ*_*U*_ = 921 nm (**c**). *Ey* component of the electric field under the condition of downward incidence at *λ*_D_ = 1315 nm (**b**) and upward incidence at *λ*_U_ = 921 nm (**d**)

Surface charge density on the silver/silica interface and *E*_*y*_ component of the electric field distribution are illustrated in Fig. 4 to reveal the SPs coupling functions. In Fig. 4a, *G*_1_ and *G*_2_ have opposite charges on their adjacent surfaces, which is similar to flat plate capacitor. Under the condition of downward incidence, *G*_1_ acts as a selector to excite SSPs at *λ*_*D*_. The periodic surface charge density distribution represents that SPPs is determined by grating constant of *G*_1_. *G*_2_ supports the ISPs induced by SPPs and performs as an emitter for transmission. *E*_*y*_ between *G*_1_ and *G*_2_ is enhanced due to the coupling between SPPs and ISPs, as shown in Fig. 4b. For upward incident condition shown in Fig. 4c, d, *G*_2_ acts as the selector and *G*_1_ acts as the emitter.

As can be seen from Fig. 4, the transmission field is periodic and nonuniform in the horizontal (*x*-axis) direction. The period *Λ* (*Λ* = 2*Λ*_1_ = 3*Λ*_2_) of the transmission filed distribution is modulated by the integral optical diode structure and satisfies 2π/*Λ* = |*g*_1_-g_2_|, here *g*_*i*_ is the grating vector of G_i_ (*i* = 1, 2). The grating diffraction efficiency is increased for the existence of SPs. The lateral wave vector *κ* of transmitted light derives from the superposition of *g*_1_ and *g*_2_:

3 $$ \kappa =\pm \frac{2\pi }{\Lambda}=\pm \left|{g}_1-{g}_2\right|, $$

And it decides the critical wavelength *λ*_*C*_ (*λ*_*C*_ = 2π/|κ|) for *T*_*D*_ ≠ *T*_*U*_. According to Eq. (3), *λ*_*C*_ is 1800 nm for our structure mentioned above, which is in good agreement with the simulation results *λ*_*C*_ = 1806 nm shown in Fig. 2. Optical diode effects appear in the range of *λ* ≤ *λ*_*C*_. According to the simulation results, the period of the integrated gratings (1800 nm) is larger than the diode operating wavelengths (1315 nm and 921 nm). Multi-order diffraction components can be obtained with light scattering from the integrated gratings. Thus, the transmission field is not uniform along the direction parallel to gratings, even when the light is transmitted to the far field.

**Fig. 5:**
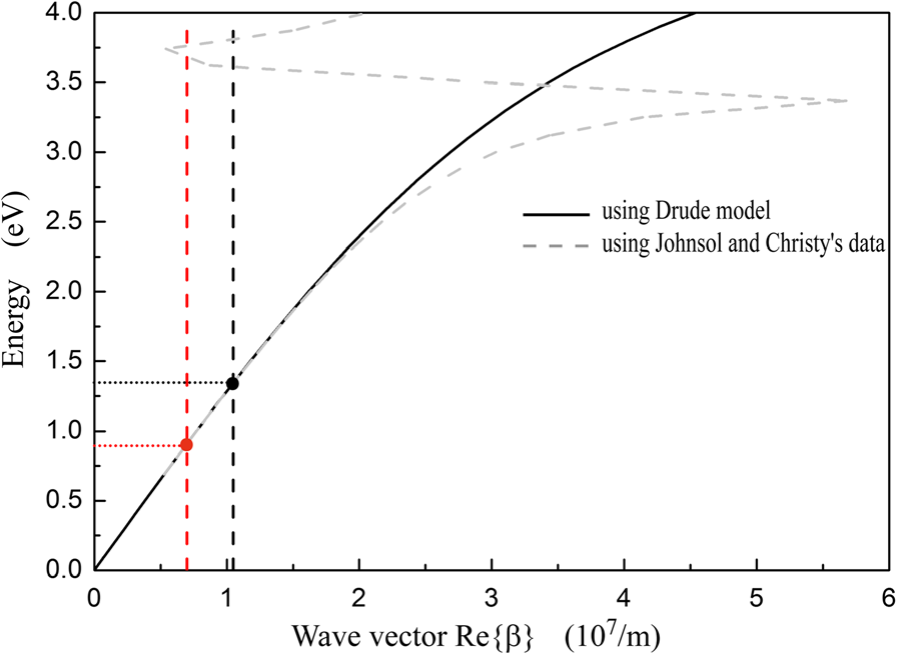
Dispersion of SPs on planar silver/silica interface calculated by using Drude model and Johnson and Christy’s optical constant data. The vertical red and black dash lines represent grating vector modulus |*g*_1_| and |*g*_2_|, respectively

SSPs of the silver grating are similar to SPs on planar silver/silica interface except that SSPs are radiative mode [37], while SPs are completely surface-bound modes. SSPs can be treated as SPs on planar silver/silica interface approximately when the slits of gratings are extremely narrow. So, the dispersion relation of SSPs can be written as [38] follows:

4 $$ \beta ={k}_0\sqrt{\frac{\varepsilon_m{\varepsilon}_d}{\varepsilon_m+{\varepsilon}_d}} $$

where *k*_*0*_ is the free space wave vector and *ɛ*_*m*_ and *ɛ*_*d*_ are the dielectric coefficient of silver and silica, respectively. The dispersion relation described by Eq. (4) is illustrated in Fig. 5. Dispersion curve calculated by using Drude model parameters [35] in this paper fits well with that calculated by using Johnson and Christy’s optical constant data sets [39] when photon energy is below 2.75 eV (*λ* > 450 nm). In Fig. 5, the vertical red and black dash lines represent |*g*_1|_ and |*g*_2_|, respectively. SSPs is excited by the grating when the vector matching condition [40] is satisfied:

5 $$ \beta ={k}_0\sin \theta \pm {Ng}_i\left(N=1,2,3\dots \right). $$

For normal incidence (*θ* = 0°), the first-order (*N* = 1) diffraction of a grating has the highest diffraction efficiency, i.e., the largest excitation efficiency for SSPs. Thus, Eq. (5) is fulfilled at the red and black points shown in Fig. 5:

6 $$ \beta =\mid {g}_i\mid . $$

In the optical diode structure, G_1_ is the selector to excite SSPs for downward incidence and *G*_2_ is the selector for upward incidence. *G*_1_ and *G*_2_ have different grating constants, so SSPs are excited at different wavelengths for reverse incident directions. In Fig. 5, the photon energy at the red point is 0.91 eV and the wavelength is 1365 nm, which is corresponding to *λ*_*D*_ (1315 nm) shown in Fig. 2. Similarly, the photon energy indicated by the black point is 1.04 eV and its wavelength is 924 nm, corresponding to *λ*_*U*_ (921 nm) in Fig. 2. As the approximation of grating to plate, the SSPs resonance wavelengths calculated by using Eq. (4) and Eq. (6) are not exactly equal to the ones simulated by using FDTD methods shown in Fig. 2.

**Fig. 6:**
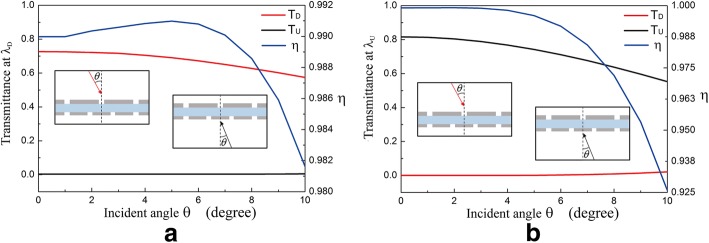
The influence of incident angle on transmittance and isolation contrast ratio at *λ*_*D*_ = 1315 nm (**a**) and *λ*_*U*_ = 921 nm (**b**)

Equation (5) indicates that incident angle *θ* influences the wave vector matching condition of grating to SSPs. With the changing of *θ*, the transmittance and isolation contrast ratio at *λ*_*D*_ (1315 nm) and *λ*_*U*_ (921 nm) are simulated and shown in Fig. 6a, b, respectively. With *θ* increasing from 0° to 10°, *T*_*D*_ at *λ*_*D*_ and *T*_*U*_ at *λ*_*U*_ decrease for the wave vector mismatching between *g*_*i*_ and SSPs. (*T*_*D*_ at *λ*_*D*_ decreases to 0 when *θ* ≈ 40° and *T*_*U*_ at *λ*_*U*_ decreases to 0 when *θ* ≈ 35°.) In incident angle range of 0° ≤ *θ ≤* 5°, *T*_*D*_ at *λ*_*U*_ and *T*_*U*_ at *λ*_*D*_ are almost 0, and *η* always keeps larger than 0.98 at both *λ*_*U*_ and *λ*_*D*_. Figure 6 demonstrates that the structure displays good optical diode effect at *λ*_D_ and *λ*_*U*_ under small-angle incidence.

### Investigation and Discussion

In this section, we investigate the influence of structure parameters on transmission spectra and isolation contrast ratio.

**Fig. 7:**
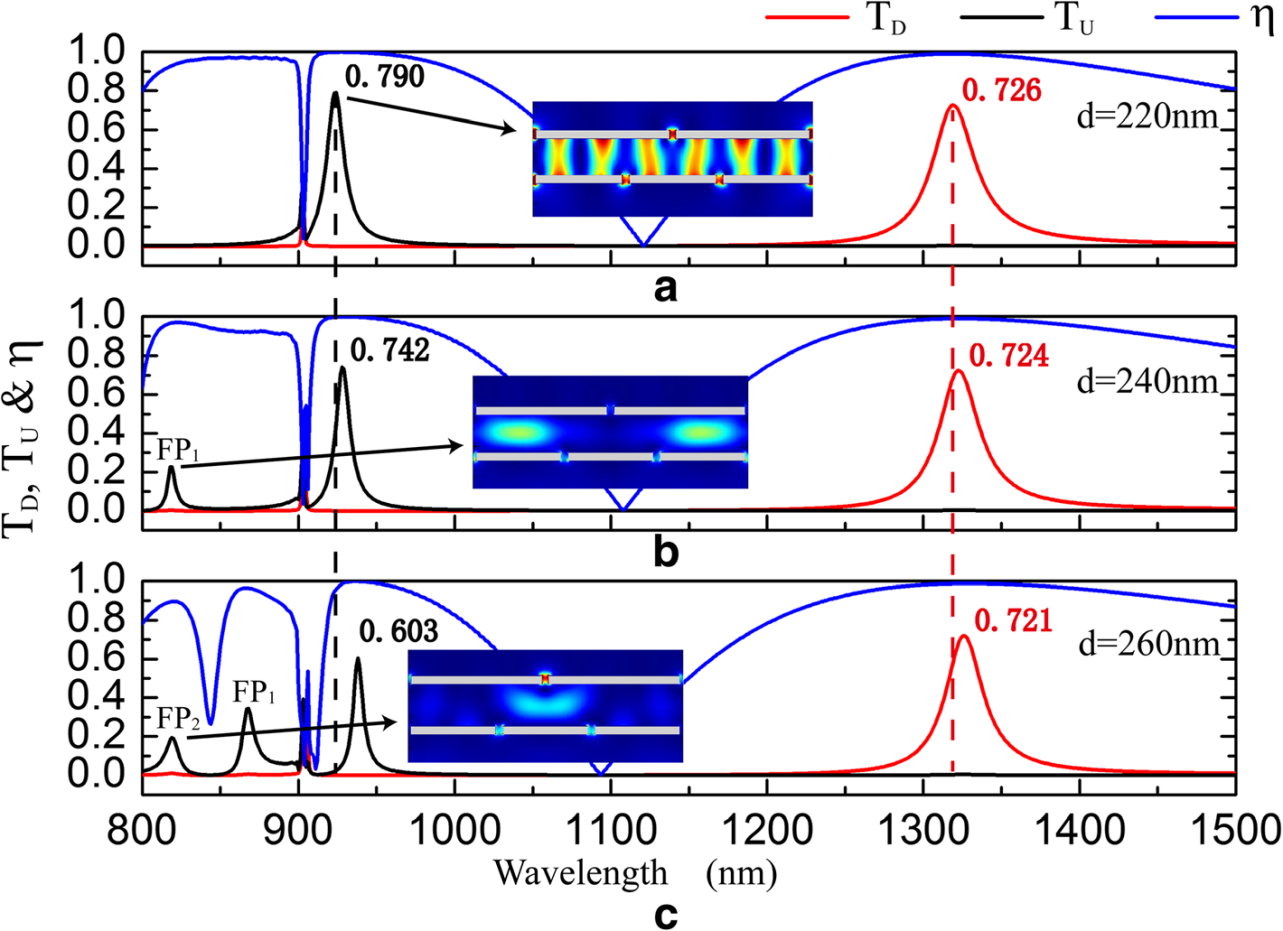
The influence of *d* on transmission spectra and the isolation contrast ratio. *d* = 220 nm (**a**), *d* = 240 nm (**b**), and *d* = 260 nm (**c**) when *s* = *h* = 50 nm, *Λ*_1_ = 900 nm, *Λ*_2_ = 600 nm, and *Δ* = 0 nm. The insets are distributions of electric field intensity |*E*|^2^ for upward transmission resonances

**Fig. 8:**
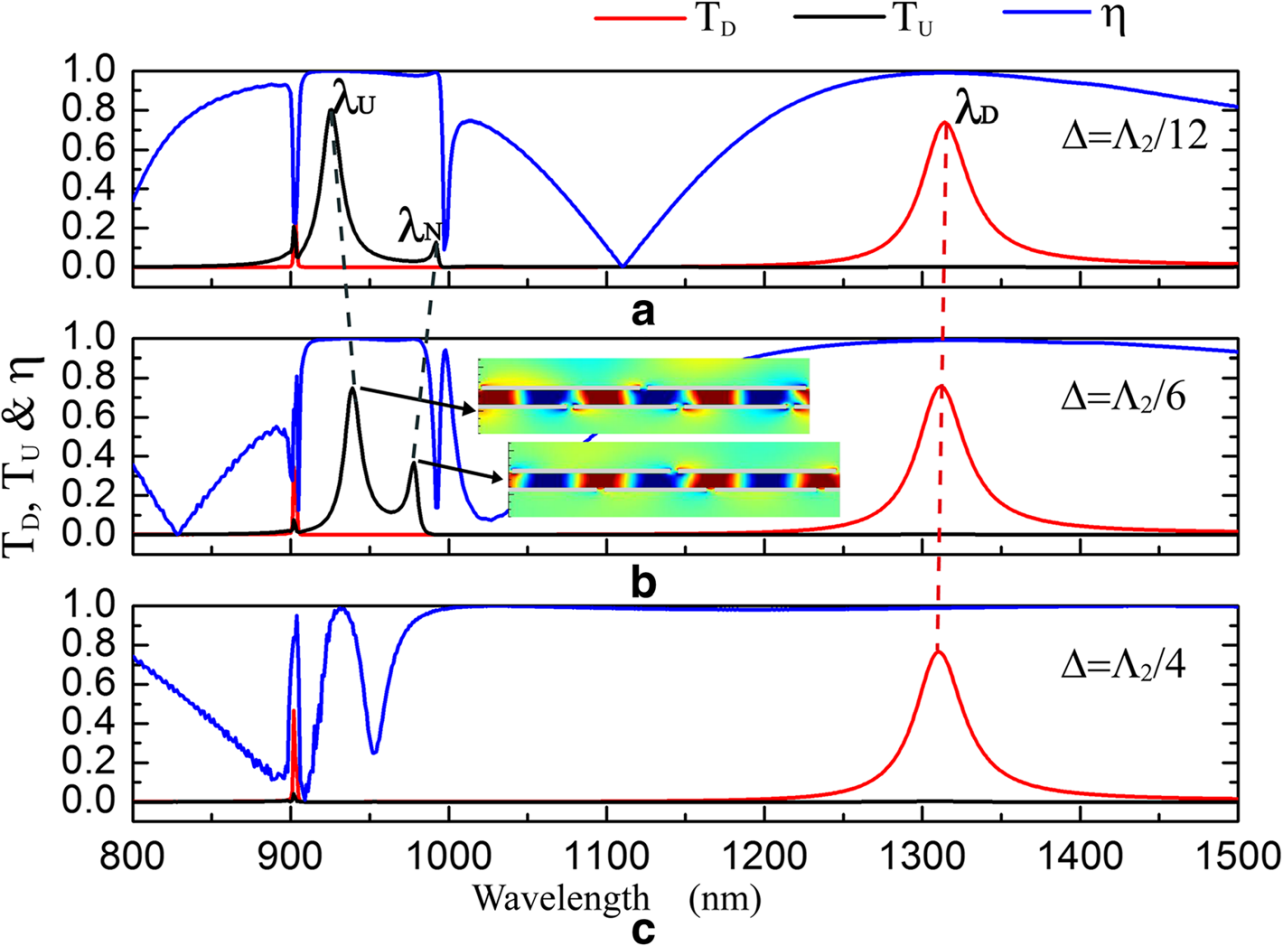
The influence of *Δ* on transmission spectra and the isolation contrast ratio. *Δ* = 50 nm = *Λ*_2_/12 (**a**), *Δ* = 100 nm = *Λ*_2_/6 (**b**), and *Δ* = 150 nm = *Λ*_2_/4 (**c**) when *d* = 200 nm, *s* = *h* = 50 nm, *Λ*_1_ = 900 nm, and *Λ*_2_ = 600 nm. The insets in (**b**) are *E*_*y*_ distributions for upward transmission resonances

The interlayer thickness *d* and gratings lateral relative position *Δ* are limited by fabrication accuracy. The influence of *d* and *Δ* on transmission spectra and the isolation contrast ratios are shown in Figs. 7 and 8, respectively. Figure 7 shows that the operating wavebands of optical diode exhibit a slight redshift when *d* increases. Meanwhile, the maximum value of *T*_*D*_ decreases very little, but the maximum value of *T*_*U*_ decreases significantly. The increase of *d* will lengthen the light transmission distance through the structure, weaken the electromagnetic interaction between *G*_1_ and *G*_2_, and impair the charge density induced at the surface of emitter. As seen in Fig. 4, charges distributed at slit corners of emitter act as electric dipoles sources of the transmission field. Charge density at slit corners of the emitter *G*_2_ (Fig. 4a) is much greater than that at slit corners of the emitter *G*_1_ (Fig. 4c), so *d* influences less on the maximum value of *T*_*D*_ than that of *T*_*U*_. Besides, with the increase of *d*, small peaks marked as FP_1_ and FP_2_ appear in *T*_*U*_ and the transmission peak of FP_1_ exhibits a large redshift. Electric filed intensity |*E*|^2^ distributions prove that FP_1_ and FP_2_ result from Fabry-Perot resonances.

As shown in Fig. 1, the optical diode structure is periodic and it has the same unit cell when *Δ = a* ± M*Λ*_2_/2 (0 nm < *a* < *Λ*_2_/2 and *M* = 0, 1, 2…). Besides, the unit cell of *Δ* = *a* is left-right flip symmetric with that of *Δ =* − *a* ± M*Λ*_*2*_/2 and they can realize the same transmission effect. So, the transmittance of the optical diode structure is affected by *Δ* as: *T*(*Δ*) = *T*(*Δ* + *Λ*_2_/2) *= T*(− *Δ* + *Λ*_2_/2). As shown in Fig. 8, optical diode effect at *λ*~921 nm turns on and off within a period of *Λ*_2_/2 as *Δ* increases. However, transmission peak of *T*_*D*_ exhibits a slight blueshift and the optical diode effect at *λ*~1315 nm is always on when *Δ* increases. Seen in Fig. 8a, a new transmission peak at *λ*_*N*_ emerges in *T*_*U*_ curve near *λ*_*U*_. When *Δ* increases from *Λ*_2_/12 to *Λ*_2_/6, the peak at *λ*_*N*_ exhibits a blueshift while the peak at *λ*_*U*_ exhibits a redshift (Fig. 8a, b). *E*_*y*_ distributions for transmission resonances at *λ*_*U*_ and *λ*_*N*_ are inserted in Fig. 8b. According to the simulation results, the resonance at *λ*_*N*_ generates because of the energy splitting. When *Δ* increases to *Λ*_2_/4, shown in Fig. 8c, *T*_*U*_ is suppressed and two transmission resonances disappear, which makes the optical diode effect turn off at *λ*~921 nm.

**Fig. 9:**
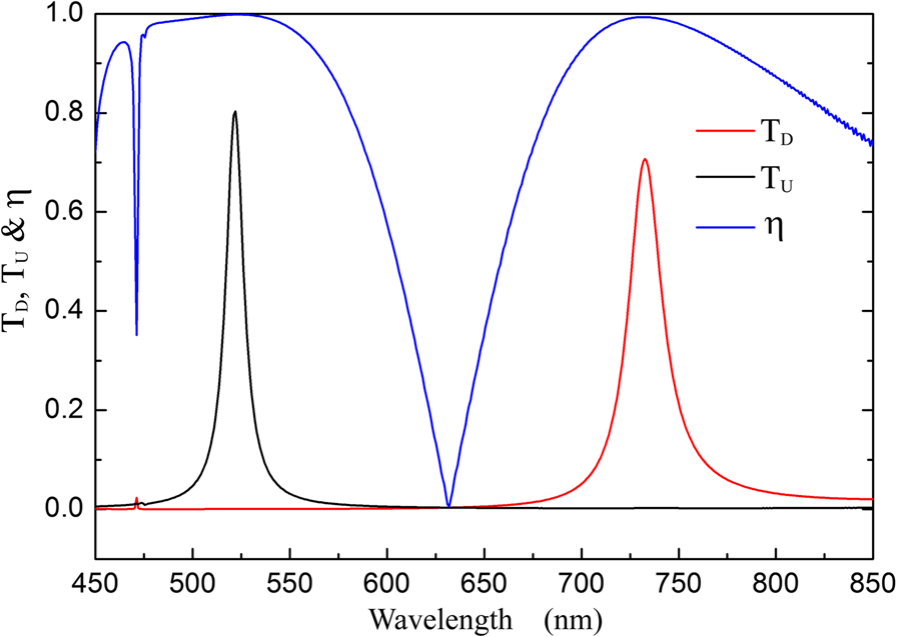
Transmission spectra and isolation contrast ratio for the optical diode structure with *d* = 100 nm, *Λ*_1_ = 450 nm, *Λ*_2_ = 300 nm, *s* = *h* = 30 nm, and *Δ* = 0 nm

According to the theory analysis, the operating waveband of optical diode can be obtained in a certain range by optimizing grating parameters. Figure 9 shows that the dichroic optical diode transmission is achieved in visible light range with structure parameters *d* = 100 nm, *Λ*_1_ = 450 nm, *Λ*_2_ = 300 nm, *s* = *h* = 30 nm, and *Δ* = 0 nm. The maximum transmittances of dichroic diode transmission wavebands are 80% (at 522 nm for upward incidence) and 71% (at 732 nm for downward incidence), and the corresponding isolation contrast ratios *η* are 0.998 and 0.993.

**Fig. 10:**
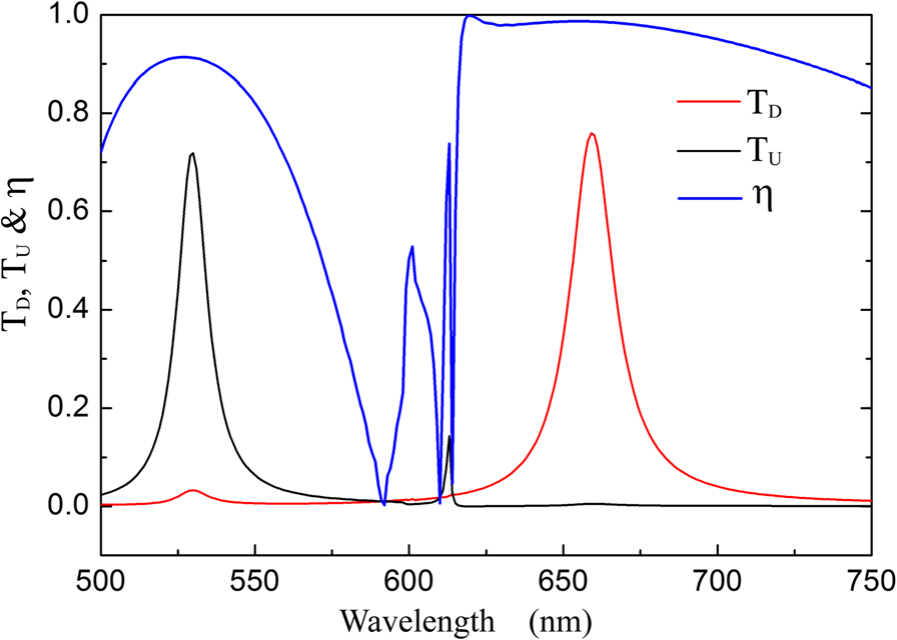
Transmission spectra and the isolation contrast ratio for the optical diode structure with the unit cell including 3 units of *G*_1_ and 4 units of *G*_2_. *d* = 100 nm, *Λ*_1_ = 400 nm, *Λ*_2_ = 300 nm, *s* = *h* = 30 nm, and *Δ* = 0 nm

Furthermore, the component of the unit cell in our structure also influences the optical diode phenomena. According to Eq. (5), the wavebands of diode effect depend on *Λ*_1_ and *Λ*_2_. In our research, we select the unit cell consisting of 2 units of *G*_1_ and 3 units of *G*_2_, i.e., 2*Λ*_1_ = 3*Λ*_2_, in order to get high transmittances and good isolation contrast ratios in the optical diode wavebands simultaneously. For example, Fig. 10 shows the dichroic transmission of the optical diode structure with its unit cell consisting of 3 units of *G*_1_ and 4 units of *G*_2_. The optical diode effects are obtain at 530 nm with *T*_*U*_ = 72% and 659 nm with *T*_*U*_ = 76%. The isolation contrast ratios at the two wavelengths are reduced to 0.912 and 0.987, because the difference of |*g*_1_*|* and |*g*_2_*|* is small and the grating acting as a selector can excite the SSPs of both gratings at different efficiencies. In addition, when *Λ*_1_ = 2*Λ*_2_, the SPs transmission resonance in the optical diode structure caused by the first-order diffraction of *G*_2_ can also be excited by the second-order diffraction of *G*_1_ for 2*g*_1_ = *g*_2_, which would reduce the isolation contrast ratio. So, the good optical diode property requires that two grating constants should have a sufficient difference and avoid the integer multiple relationship.

## Conclusions

The dichroic optical diode transmission based on SPs is realized in our structure, which consists of two dislocated parallel silver gratings and a silica interlayer. The first illuminated metallic grating selects the transmission waveband by exciting SSPs, and the other metallic grating emits electromagnetic energy forward through the surficial electrons oscillations. When the incident direction of light is reversed, the roles of two gratings exchange and another optical diode transmission waveband appears. The optical isolation ratio can almost reach up to 1. Optical diode transmission wavebands can be adjusted to be in different regions by changing the structure parameters. The optical diode operating wavebands and transmittance are independent of the incident intensity. The thickness of the structure is only a few hundred nanometers. These properties of our structure provide a wide range of applications in integrated circuits.

## Abbreviations

ISPs: Induced surface plasmons.
SPs: Surface plasmons
SSPs: Structured surface plasmons
